# Decoding Spatial Versus Non-spatial Processing in Auditory Working Memory

**DOI:** 10.3389/fnins.2021.637877

**Published:** 2021-02-19

**Authors:** Mira Erhart, Stefan Czoschke, Cora Fischer, Christoph Bledowski, Jochen Kaiser

**Affiliations:** ^1^Institute of Medical Psychology, Medical Faculty, Goethe University Frankfurt am Main, Frankfurt am Main, Germany; ^2^International Max Planck Research School – Translational Psychiatry (IMPRS-TP), Max Planck Institute of Psychiatry, Munich, Germany; ^3^Brain Imaging Center, Medical Faculty, Goethe University Frankfurt am Main, Frankfurt am Main, Germany

**Keywords:** working memory, functional magnetic resonance imaging, multivoxel pattern analysis, auditory, pitch, location, searchlight analysis

## Abstract

**Objective:**

Research on visual working memory has shown that individual stimulus features are processed in both specialized sensory regions and higher cortical areas. Much less evidence exists for auditory working memory. Here, a main distinction has been proposed between the processing of spatial and non-spatial sound features. Our aim was to examine feature-specific activation patterns in auditory working memory.

**Methods:**

We collected fMRI data while 28 healthy adults performed an auditory delayed match-to-sample task. Stimuli were abstract sounds characterized by both spatial and non-spatial information, i.e., interaural time delay and central frequency, respectively. In separate recording blocks, subjects had to memorize either the spatial or non-spatial feature, which had to be compared with a probe sound presented after a short delay. We performed both univariate and multivariate comparisons between spatial and non-spatial task blocks.

**Results:**

Processing of spatial sound features elicited a higher activity in a small cluster in the superior parietal lobe than did sound pattern processing, whereas there was no significant activation difference for the opposite contrast. The multivariate analysis was applied using a whole-brain searchlight approach to identify feature-selective processing. The task-relevant auditory feature could be decoded from multiple brain regions including the auditory cortex, posterior temporal cortex, middle occipital gyrus, and extended parietal and frontal regions.

**Conclusion:**

In summary, the lack of large univariate activation differences between spatial and non-spatial processing could be attributable to the identical stimulation in both tasks. In contrast, the whole-brain multivariate analysis identified feature-specific activation patterns in widespread cortical regions. This suggests that areas beyond the auditory dorsal and ventral streams contribute to working memory processing of auditory stimulus features.

## Introduction

A central question in neuroscientific research on human working memory (WM) concerns the brain regions involved in the processing of memory contents. A recent review of electrophysiological and neuroimaging studies has suggested a distributed representation of memorized information across the brain (Christophel et al., 2017). Consistent with the view that elementary stimulus features are maintained in sensory regions (Pasternak and Greenlee, 2005), multivoxel pattern analysis (MVPA) has revealed a persistent stimulus-specific activity in the sensory cortex (Harrison and Tong, 2009; Serences et al., 2009; Christophel et al., 2012; Riggall and Postle, 2012; Emrich et al., 2013; Peters et al., 2015) and in specialized areas along the processing hierarchy (Riggall and Postle, 2012; Christophel and Haynes, 2014). However, more recent studies have demonstrated stimulus-selective activation patterns also in the frontal and parietal regions (Christophel et al., 2015; Ester et al., 2015; Peters et al., 2015). While sensory recruitment seems to be an important feature of stimulus processing in WM, the representation of stimulus-specific information in the fronto-parietal cortex may depend on task demands (Lee et al., 2013; Bettencourt and Xu, 2016; Christophel et al., 2017; Xu, 2017).

Compared with the visual domain, relatively few studies have investigated WM processing of acoustic stimulus features. Here, a prominent distinction has been made between sound pattern and sound identity on the one hand and its spatial location on the other hand. Separate ventral and dorsal pathways have been proposed for the perceptual processing of non-spatial and spatial features, respectively (Rauschecker, 1998; Rauschecker and Tian, 2000). Electrophysiological and anatomical studies in monkeys have suggested that the ventral, pattern processing stream involves the anterior auditory cortex and anterior temporal and inferior frontal cortices, whereas the dorsal, space processing stream includes the posterior auditory cortex, and posterior parietal and superior frontal regions (Romanski et al., 1999; Tian et al., 2001). Both neuropsychological and functional imaging work in humans has provided converging evidence for this division (Alain et al., 2001; Clarke et al., 2002; Arnott et al., 2004; Altmann et al., 2007).

Concerning WM, hemodynamic activation differences in the regions of the putative auditory dorsal and ventral streams have been associated with the memorization of spatial and non-spatial sound features, respectively. Contrasting the processing of pitch versus location of noise bursts in a match-to-sample task with a brief delay revealed increased fMRI activation in the auditory cortex and inferior frontal cortex for pitch and in the posterior temporal, parietal, and superior frontal cortices for location (Alain et al., 2001). Similar results were obtained for WM processing of voice identity versus location (Rämä et al., 2004), for the processing of sound identity defined by the temporal position of a gap of silence in a noise sound compared with location processing (Arnott et al., 2005), and for n-back tasks requiring the categorization of natural sound identity versus location (Alain et al., 2008, 2018). Preferential processing of spatial sound features in the posterior regions and of non-spatial features in anterior brain areas has also been found in magnetoencephalography (MEG) studies focusing on the spectral activity in the gamma band (Lutzenberger et al., 2002; Kaiser et al., 2003, 2009).

Direct contrasts between activations during spatial versus non-spatial auditory WM have thus supported the segregated processing of both types of information in regions along the proposed pathways, but not in the early auditory cortex. Moreover, comparisons with baseline have typically shown a large overlap of activations for both tasks (e.g., Alain et al., 2001). Multivariate approaches offer the possibility to detect distinct activation patterns also in regions that show an equally strong overall activity for different contents or tasks (Kriegeskorte et al., 2006; Haynes, 2015). Several fMRI studies have used multivariate analyses to decode stimulus-selective patterns during processing of sound patterns in auditory WM. Linke et al. (2011) assessed pattern similarity between four blocks of frequencies during a delayed match-to-sample task for pairs of pure tones. Frequency-selective responses during maintenance were found in Heschl’s gyrus only. Using MVPA, the same group found sound identity coding of complex environmental sounds in the auditory cortex, Heschl’s gyrus, and middle temporal cortex (Linke and Cusack, 2015). Kumar et al. (2016) decoded low- versus high-frequency tones during WM maintenance in the auditory cortex and left inferior frontal cortex. Most recently, the WM representation of amplitude-modulated sounds was decoded in regions including the superior temporal gyrus and precentral cortex (Uluc et al., 2018). In summary, MVPA studies have provided evidence for an involvement of the auditory cortex in the WM processing of auditory pattern information. However, we are not aware of any fMRI work attempting to decode auditory spatial versus non-spatial WM processing.

Using MEG broadband signals, we have tested task selectivity during an auditory WM paradigm, where a cue indicated for each trial whether sound lateralization or pitch was the task-relevant feature (Peters et al., 2016). Applying linear discriminant functions revealed task-selective signal patterns throughout the trial, including the pre-encoding, encoding, and maintenance phases. Temporal cross-decoding suggested that task-specific codes were established at the beginning of trial and reactivated during subsequent stimulus processing in WM. As this study focused on the temporal dynamics of task-selective signal patterns, data from all MEG sensors were combined in the analysis. We therefore obtained only limited information about the topography of these patterns.

In contrast, the current study aimed at identifying brain regions whose fMRI signal patterns distinguish between spatial and non-spatial auditory WM processing. We did not focus on any specific subprocess of WM but assessed the attentional selection of pitch versus location across the different phases of a WM task including encoding, maintenance, and retrieval. Participants performed a delayed match-to-sample tasks with abstract sounds characterized by both their central frequency (pitch) and interaural time delay (location). In separate blocks, either the spatial or non-spatial feature was task relevant. We expected decodability of the task-relevant feature in regions along the putative auditory dorsal and ventral streams including the early auditory cortex.

## Materials and Methods

### Participants

Forty-two healthy adults took part in a behavioral screening session (29 females; mean age 22.7 years, SD = 4.0 years). Fourteen participants were excluded after behavioral testing for one of the following reasons: reporting the task as being very difficult (two participants), detection threshold for location changes exceeded 70° (six participants), poor accuracy in reproducing pitch or location from memory (three participants), or dropout prior to the first fMRI session (three participants). The remaining 28 subjects (20 females, mean age 21.0 years, SD = 5.4 years) completed three fMRI sessions. Sixteen participants played or had played an instrument, four were choir members or took singing lessons, and seven subjects took dancing lessons. The mean musical experience was 5.5 years (SD = 5.1 years). Subjects reported normal hearing and no diseases of the auditory system and met MR imaging requirements. All participants provided their written informed consent to participate in this study and received a remuneration of €10/h. The study was approved by the ethics committee of the Goethe University medical faculty.

### Stimuli

Sample sounds were two-dimensional feature combinations of a complex sound and a spatial location. Each sound was composed of a fundamental frequency and two harmonics. Different pitch values were obtained by varying the fundamental frequencies in a range from 286.41 to 451.15 Hz in six steps of 0.09 log(10) Hz in logarithmic space. All three components were band-pass filtered to a bandwidth of 1/10 octave to smoothen the sound perception. Spatially localized sounds were created by introducing an interaural time difference (ITD). The following ITDs were used for the sample sounds: 0.53, 0.34, and 0.12 ms, corresponding roughly to lateralization angles of 64°, 39°, and 13° from the center to the left and to the right, respectively. By combining six pitch with six location values, 36 different sample sounds could be generated. Probe sounds were created in an adaptive manner as described below in the section “Procedure.” Stimuli were processed with an external soundcard (Fireface UC, 192-kHz sampling rate, RME, Haimhausen, Germany). For behavioral testing outside the scanner, they were presented via headphones (K271 MkII, AKG, Vienna, Austria), whereas we used MRI-compatible noise-canceling headphones (OptoActive, Optoacoustics Ltd., Mazor, Israel) for stimulus presentation in the MR scanner. Stimulus construction and timing were controlled with Matlab R2012b (MathWorks, Inc., Natick, MA, United States) and the Psychophysics Toolbox (Brainard, 1997).

### Procedure

Participants performed an auditory delayed match-to-sample task, in which the task-relevant stimulus feature (location or pitch) alternated between recording blocks while stimulation did not differ between tasks. The task-relevant feature (location or pitch) was cued by a colored fixation circle that appeared at the beginning of each block and stayed on the screen throughout the block. The circle was either yellow or blue. The assignment of color to the task-relevant feature was balanced across subjects.

**FIGURE 1:**
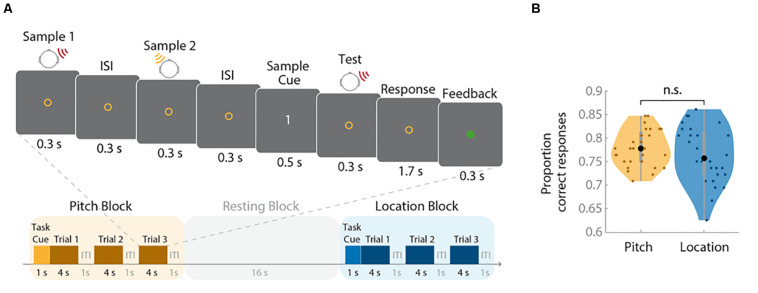
Trial structure and behavioral results. **(A)** The upper panel illustrates the structure of an individual trial (duration: 4 s); the lower panel depicts the sequence of task and resting blocks (duration: 16 s each). **(B)** Proportions of correct responses for each task are shown as violin plots. Small colored dots represent the values of individual participants, the larger black dots reflect the group mean, the bold gray bars indicate the interquartile range, the gray thin bars represent the whiskers with maximum 1.5 interquartile range, and shading represents the density trace. The performance did not differ between tasks (n.s., not significant).

The trial structure is depicted in Figure 1A. One second after the onset of the colored task cue, each trial started with the presentation of the first sample stimulus for 300 ms. After an inter-stimulus interval of 300 ms, the second sample sound was presented for another 300 ms. Three hundred milliseconds after the second stimulus, a numeric cue (digits “1” or “2”) appeared for 500 ms, indicating whether the first or second stimulus had to be compared with the upcoming test tone. After cue presentation, the probe stimulus appeared immediately for 300 ms. During the subsequent 1.7-s response phase, participants indicated whether or not this stimulus matched the target sound on the cued feature dimension (location or pitch) by pressing a trackball button (Current Designs, Philadelphia, United States). A match was indicated by a left click and a non-match by a right click. If participants did not respond within 2 s, the trial was recorded as incorrect. Feedback was provided via a 300-ms color change of the fixation circle. Changes to green or red indicated correct or incorrect responses, respectively. The duration of the inter-trial interval (ITI) was 1 s. There was one run during each of the three fMRI sessions. Each run contained 16 blocks (eight per feature), resulting in a total number of 72 trials per feature. The target feature alternated sequentially between blocks. The target feature of the first block and the color-target feature assignment were cross-balanced. Half of the trials for each feature were match trials (i.e., target and test stimuli were identical).

To obtain comparable difficulty levels for pitch and location tasks, probes were generated according to an adaptive procedure. The distance between the sample feature and the probe feature varied depending on each subject’s performance. In non-match trials, the difference between the target and test stimuli was controlled by a one-up/two-down staircase procedure (Levitt, 1971). The initial distance between target and probe was set to 80° for the location task and 0.04 log(10) Hz for the pitch task. Every time a subject failed in a non-match trial, the distance was increased by 5° for the location and by 0.02 log(10) Hz for the pitch task. The distance was decreased again if subjects achieved two consecutive correct non-matches. This aimed at controlling the performance in non-match trials so that the probability for a correct non-match response was 70.7% for each subject. The staircases ran continuously across all four sessions, i.e., the behavioral session and three fMRI sessions. On each trial, pitch and location were drawn randomly without replacement for both stimuli, with the restriction that laterality was counterbalanced per run and target feature. Serial position and laterality of the target stimulus were also counterbalanced across runs and features.

There was one initial behavioral testing session during which MR scanner noise was played as a background sound. As the current experiment was combined with another study that will be reported elsewhere, fMRI data acquisition was distributed across three scanning sessions. All sessions took place on separate days.

### fMRI Data Acquisition

fMRI data were collected with a 3-T Magnetom Prisma scanner (Siemens, Erlangen, Germany) at the Brain Imaging Center of the Goethe University of Frankfurt medical faculty. We used a 64-channel head coil. Structural scans were acquired using GeneRalized Autocalibrating Partially Parallel Acquisition (GRAPPA) with a spoiled-gradient T1-weighted sequence that yielded a 1-mm^3^ resolution. The acquisition orientation was sagittal with a repetition time (TR) of 1,000 ms and an echo time (TE) of 2.52 ms. Field of view was 256 mm. Functional scans started with a 5-s period without any stimuli to account for changes in the signal until brain magnetization stabilized. These scans were excluded from the analysis. Whole-brain echo-planar images (EPIs) were acquired in 51 transverse slices with TE = 30 ms and TR = 1,000 ms. Image parameters were a 64 × 64 image matrix, 90° flip angle, 192 mm field of view, and 3 × 3 × 2 mm slice thickness. Images were acquired interleaved.

### fMRI Data Preprocessing

Preprocessing was performed using Statistical Parametric Mapping 12 (SPM, UCL Queen Square Institute of Neurology, 2014) and the FSL toolbox topup (Andersson et al., 2003). As a first step, data were distortion corrected using the topup toolbox implemented by Smith et al. (2004). Data were then motion corrected to the last volume of each session. Rigid body spatial transformation parameters estimated during this step were added as regressors to the general linear model (GLM) later on. The last volume of each session and the first volume of the subsequently measured correction scan with reversed phase-encoding direction were used to estimate the susceptibility-induced off-resonance field. All other volumes were corrected using this field map. As a second step, data were motion-corrected by adjusting all images to the mean functional image using a least-squares fit. Subsequently, a manual anterior commissure to posterior commissure (AC-PC) alignment for structural and motion-corrected functional images was performed before data were coregistered to their structural scans. For the multivariate analysis, the coregistered images were used, whereas images were normalized and smoothed for the univariate approach. Segmented gray matter fields were used to normalize the individual subject’s space to the Montreal Neurological Institute (MNI) reference space. This approach reduces confounding with non-brain tissue during normalization. Finally, all images were smoothed using a 6-mm Gaussian smoothing kernel.

#### Univariate Analysis

To identify brain regions showing a stronger blood oxygen level-dependent (BOLD) effect during the processing of spatial versus non-spatial auditory stimulus features, contrasts between both tasks were calculated. A GLM with eight regressors was modeled. The first two regressors modeled the blocks for the different conditions (pitch and location tasks). Regressors 3 to 8 contained motion correction parameters. All conditions were convolved with the hemodynamic response function (HRF). After model estimation, two opposing contrasts were calculated to test relative activation increases for the location compared with the pitch task and vice versa.

#### Multivariate Analysis

For the multivariate analysis, a second GLM on the single-subject level was established. It used the same eight regressors as for the univariate GLM convolved with the HRF. However, functional images before normalization and smoothing were used, thus preserving the native space of each individual subject. Based on cross-validated multivariate analysis of variance (cvMANOVA; Allefeld and Haynes, 2014), we performed a searchlight analysis with a radius of three voxels (∼9 mm; ∼123 voxels of 3 × 3 × 3 mm) restricted to whole-brain-mask voxels created by SPM during model estimation. cvMANOVA quantifies the differences in BOLD activity patterns attributable to an experimental condition on the single-subject level and expresses it in the pattern distinctness measure D. D is an interpretable, cross-validated, standardized effect size. Cross-validation is based on a leave-one-run-out procedure and limits D to zero if the experimental conditions do not elicit differential voxel patterns. Note that we applied cvMANOVA to compare the activity patterns of only two feature classes. In this case, D is comparable with the Mahalanobis distance, which offers a reliable expression of pattern dissimilarity (e.g., Kriegeskorte et al., 2006). cvMANOVA has several advantages over other MVPA approaches (Allefeld and Haynes, 2014; Sohoglu et al., 2020). It relies on neither the specific classifier and its parameters nor the assignment of the data to a training and test set but directly quantifies the explained variance by a specific condition derived from the distinctness in activity patterns. Additionally, it is more sensitive than classification accuracy (Christophel et al., 2018) because it considers the spatial structure of the noise by relativizing the multivoxel signal for a given condition by the noise covariance between voxels (Allefeld and Haynes, 2014). As recommended by Allefeld and Haynes (2014), pattern distinctness D was standardized by the number of voxels within the searchlight to correct for inaccuracy caused by varying numbers of within-mask voxels at the borders of the brain mask. For the purpose of statistical inference, the resulting maps of standardized D values were normalized to MNI space and smoothed with a 6-mm Gaussian smoothing kernel.

### Statistical Analysis

#### Univariate Analysis

The contrasts revealed by the univariate analysis for the individual subjects were aggregated for the group in a random-effects analysis. Two 1-sample *t*-tests, one for each contrast, were conducted to identify brain regions with a stronger activation during the processing of spatial versus non-spatial auditory content. Family-wise error (FWE) correction at *p* < 0.05 was applied to account for multiple comparisons.

#### Multivariate Analysis

Single-subject D-maps of searchlight results were analyzed on the group level using a one-sample non-parametric permutation test (Nichols and Holmes, 2002) implemented in the Statistical non-Parametric Mapping (SnPM) toolbox^1^. In this procedure, the sign of the of pattern distinctness D for each subject and voxel is randomly flipped, and thus, a null distribution of voxel-wise pattern distinctness is generated across multiple iterations. We ran 10,000 iterations with a 6-mm variance smoothing kernel revealing pseudo *t*-values. Results were thresholded voxel-wise at *p* < 0.05 (FWE-corrected).

## Results

### Behavioral Performance

The mean minimal distance between two feature characteristics (threshold) required to distinguish them was 0.027 × log(10) Hz (SD = 0.025 × log(10) Hz) for pitch and 47.46° (SD = 21.81°) for location. The proportions of correct responses for the location and pitch tasks are depicted in Figure 1B. Correct response rates did not differ between conditions, *t*(27) = −1.06, *p* = 0.30. To check whether the staircase procedure worked, the probability for correct non-matches for the two features across all sessions was tested against 70.7%, representing the estimated stable threshold derived from the staircase procedure. The difference was non-significant for both pitch blocks (*t*(27) = 1.70, *p* = 0.101) and location blocks (*t*(27) = 1.80, *p* = 0.083). These results indicate that the staircase procedure was successful in matching the task difficulty of the pitch and location blocks.

### Univariate Analysis

**FIGURE 2:**
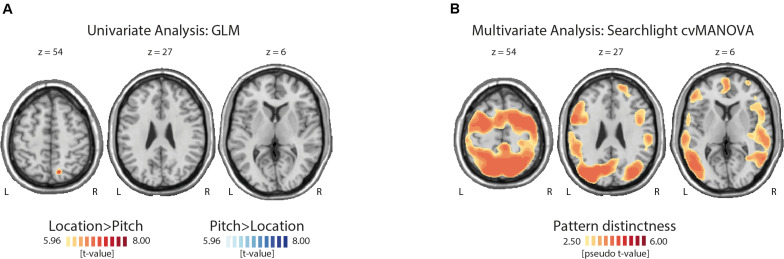
Topographies of uni- and multivariate fMRI analysis results. **(A)** The univariate comparison yielded only one significant cluster in the superior parietal cortex with a higher activation during the location compared with the pitch task. **(B)** This figure illustrates some of the regions where the cross-validated multivariate analysis of variance (cvMANOVA) searchlight analysis showed a significant decoding of the task-relevant feature (pitch versus location). For a full list of regions, see Table 1.

We used a whole-brain analysis to determine the cortical regions showing stronger activations during auditory spatial versus non-spatial WM processing. More specifically, we asked where the BOLD signal associated with pitch processing during an auditory WM task exceeded the signal during the processing of location information and vice versa. There was no region where the activation during the non-spatial task exceeded the activation elicited by the spatial task after correcting for multiple comparisons. In contrast, a cluster in the right superior parietal lobe (MNI coordinates: *x*: 14, *y*: −68, *z*: 54; *z*-value 5.16, peak-level inference: *p* = 0.002, FWE-corrected, cluster size 89 voxels) showed a stronger activation for location than pitch blocks (Figure 2A).

### Multivariate Analysis

Significant pattern distinctness was observed in 18 uni- and bilateral cortical regions (Figure 2B). Lateralization, pseudo *t*-values, cluster sizes, and coordinates of cluster peaks are listed in Table 1. We found distinct activity patterns in regions along the putative auditory dorsal and ventral pathways including the bilateral auditory cortex, the left inferior as well as right middle and superior temporal gyri, the right superior parietal lobe, the precentral gyrus, and the right superior, left inferior, and right middle frontal gyri. Moreover, we found significant pattern distinctness in several regions less strongly associated with auditory processing like the bilateral middle occipital and bilateral inferior parietal lobes, the left supramarginal gyrus, the right precuneus, the left cingulate gyrus, and the bilateral postcentral gyrus.

**TABLE 1:** Results of the multivariate searchlight analysis.

		**MNI coordinates**				
**Anatomical region**	**Hemisphere**	***x***	***y***	***z***	**Cluster size**	**Pseudo *t*-value**
**Temporal cortex**						
Primary auditory cortex	L/R	−44/−44	−20/18	12/−32	29/55	2.80/2.59
Superior temporal gyrus	R	54	−22	−2	800	3.06
Middle temporal gyrus	R	58	−58	2	749	3.16
Inferior temporal gyrus	L	−50	−72	−26	1,279	3.55
**Frontal cortex**						
Precentral gyrus	L/R	−46/46	−4/4	38/30	1,632/1,252	3.46/3.04
Superior frontal gyrus	L/R	0/20	10/56	52/26	789/392	3.39/2.67
Middle frontal gyrus	L	36	58	−4	337	2.81
Inferior frontal gyrus	L	−56	12	18	1,291	3.36
Medial frontal gyrus	R	4	56	−8	828	3.18
Cingulate gyrus	L	−18	−4	48	1,581	3.45
**Parietal cortex**						
Postcentral gyrus	L/R	−60/32	−16/−30	32/50	555/911	2.67/3.04
Superior parietal lobe	R	18	−40	66	1,487	3.09
Inferior parietal lobe	L/R	−52/62	−38/−24	30/30	1,046/1,019	3.33/3.12
Supramarginal gyrus	L	−40	−36	38	1,368	3.46
Precuneus	R	16	−66	38	4,022	3.79
**Occipital cortex**						
Middle occipital gyrus	L/R	−28/34	−88/−78	22/18	1,150/552	2.94/3.51

*R, right; L, left; cluster size indicates N voxels. MNI, Montreal Neurological Institute.*

## Discussion

While brain imaging work has demonstrated the involvement of sensory and higher-level regions in WM processing of visual stimulus features, relatively little evidence exists on the auditory domain. The present study aimed at identifying brain regions showing selectivity for the processing of spatial versus non-spatial sound attributes in auditory WM. Using identical stimuli characterized both by a specific frequency composition and ITD, participants were asked to memorize either the spatial or non-spatial feature in separate task blocks. The univariate contrast between blocks yielded no differences in the early auditory cortex. We observed clusters with a stronger fMRI activity for location than pitch in the superior parietal cortex, which has been found to be involved in visual and auditory spatial WM (Hamidi et al., 2008; Michalka et al., 2016). In contrast, we found no significant clusters with an enhanced activity for pitch compared with location memory processing. In contrast, the multivariate analysis showed distinct hemodynamic response patterns for spatial versus non-spatial auditory WM in widespread cortical areas. As hypothesized, fMRI signal patterns differed between task-relevant features in brain regions thought to form part of the auditory processing streams like the auditory cortex, inferior and superior parietal regions, and inferior and superior frontal cortices. In addition, the present whole-brain searchlight analysis showed task decodability in regions not specialized in auditory processing including the occipital cortex, and precuneus or postcentral gyri. These findings thus suggest differential activation patterns during WM processing of spatial versus non-spatial sounds in widespread cortical regions, most of which were not detectable with a univariate analysis.

The multivariate analysis thus supported the segregated processing of auditory spatial versus non-spatial stimulus features in brain regions along the putative auditory “what” and “where” pathways, ranging from the early auditory cortex via further temporal regions to the frontal and posterior parietal cortices, respectively (Rauschecker, 1998; Romanski et al., 1999; Arnott et al., 2004; van der Heijden et al., 2019). While our univariate results are in line with previous auditory WM research (Alain et al., 2001, 2008; Arnott et al., 2005), the decoding analysis demonstrated also the involvement of early sensory regions in the differential processing of both types of auditory information. The decodability of auditory memory contents in the auditory cortex as well as in auditory ventral stream regions is consistent with multivariate studies that investigated the WM processing of sound pattern or identity (Linke et al., 2011; Kumar et al., 2016; Uluc et al., 2018). By contrasting blocks with spatial versus non-spatial auditory WM tasks, the present study extended these findings by showing that sound feature-specific response patterns can be found also in the auditory dorsal pathway.

Our findings are compatible with the sensory recruitment account of WM, postulating an involvement of sensory regions in WM processing (Pasternak and Greenlee, 2005; Scimeca et al., 2018). The absence of activations in the low-level auditory cortex found in both univariate fMRI (Alain et al., 2001, 2008; Arnott et al., 2005) and MEG studies (reviewed by Kaiser, 2015) could be attributable to the fact that maintenance-related activations are distributed across the sensory cortex, preventing univariate methods from detecting spatially contiguous activation clusters (Riggall and Postle, 2012). Here, multivariate approaches offer additional insights by identifying stimulus- or task-specific activation patterns across multiple voxels within a given region (Haxby et al., 2014).

The fact that MVPA is a sensitive method for examining differences between neural activation patterns that cannot be detected using classical mass-univariate analysis might also account for the striking discrepancy between the present paucity of univariate effects and the topographically widespread decodability of the task-relevant feature. Multivariate, information-based approaches exploit the high spatial resolution of fMRI more effectively than univariate, activation-based analyses and are better suitable for detecting response patterns with a fine-grained spatial distribution (Kriegeskorte et al., 2006; Haynes, 2015). For example, an early MVPA study (Haxby et al., 2001) demonstrated that visual object categories could be decoded from the ventral temporal cortex beyond regions identified as category-selective by univariate analyses; e.g., images of faces could be discriminated outside the fusiform face area. fMRI classification also revealed sustained activity patterns in the visual cortex that predicted the contents of visual WM despite low overall levels of activation in this region (Harrison and Tong, 2009). Similarly, multivariate methods served to identify brain regions responding selectively to more abstract task features like stimulus-response mapping rules, whereas univariate comparisons did not yield consistent effects (Woolgar et al., 2011). The present results are thus compatible with previous research showing that decoding methods can reveal information present in the brain activity that is undetectable by univariate methods.

Unexpectedly, we found decodability of the task-relevant auditory feature also in a number of brain regions not typically associated with auditory processing. Here, we can only speculate about their possible role in the present auditory WM task. It is conceivable that regions associated with visuo-spatial processing like the middle occipital gyrus (Renier et al., 2010) or precuneus (Frings et al., 2006; Müller et al., 2018) are also involved in auditory spatial WM. This would be consistent with evidence from experimental psychology suggesting that auditory and visual locations are stored in a common memory (Lehnert and Zimmer, 2006). Spatial versus non-spatial sounds could also be associated with different levels of visual imagery involving the occipital cortex (Vetter et al., 2014). Moreover, we cannot exclude the possibility that the colored cues indicating the task-relevant stimulus dimension may have contributed to the effect in the visual cortex. The sparse evidence concerning the role of pre- and postcentral gyri in auditory WM suggests an involvement of these regions in target processing for both spatial and non-spatial tasks (Alain et al., 2008).

An alternative explanation for the present decoding results outside auditory processing regions would be differences in task difficulty between the auditory spatial and non-spatial WM tasks. They may have given rise to differential activation patterns in regions belonging to the fronto-temporal attention network like the superior frontal and intraparietal cortices (Corbetta and Shulman, 2002) or to the default-mode network like the cingulate cortex or precuneus (Raichle, 2015). However, such effects might have also been detectable in the univariate analysis. Moreover, the anterior insula and anterior cingulate cortex, which are known to respond strongly to increased task demands (Duncan, 2010; Lamichhane et al., 2016), showed neither activation differences between the present conditions nor distinct feature-specific signal patterns. However, as the increased sensitivity of MVPA may come at the price of reduced specificity (Woolgar et al., 2014), care has to be taken to avoid possible confounds such as differences in task difficulty at the level of single participants (Todd et al., 2013; Haynes, 2015). Here, we addressed this issue by using an adaptive procedure to ensure comparable difficulty levels between tasks. In summary, it seems unlikely that the present findings are attributable to differences in task difficulty.

In general, the interpretability of the present decoding results is limited. Our study design does not allow conclusions about which auditory feature drove the effect in a given region. There is fMRI evidence for a processing of sound identity in the auditory cortex (Linke et al., 2011; Kumar et al., 2016) and middle temporal (Linke and Cusack, 2015) and inferior frontal cortices (Alain et al., 2001; Kumar et al., 2016) and of location in the parietal (Alain et al., 2008; Arnott et al., 2005) and superior frontal regions (Alain et al., 2008). A different study design enabling the decoding of different feature values within each dimension is required to determine whether fMRI signals in a given region contain information particularly for sound features.

The block design of the present study and its temporal structure with a brief delay phase represent further limitations. They made it impossible to attribute our findings to any particular subprocess of WM such as encoding, maintenance, or retrieval. Therefore, any interpretation of the present findings as specific to WM has to be treated with caution. It is well conceivable that the present decoding results reflect attentional orienting or task sets related to the processing of pitch versus location of acoustic stimuli during WM encoding, maintenance, and retrieval. In fact, our previous MEG study on spatial versus non-spatial auditory WM has shown that such task sets are established on a trial-by-trial basis and can be decoded during the pre-encoding phase as well as during the actual task (Peters et al., 2016). To obtain conclusive evidence about WM-specific processing, a different task design with, e.g., longer delay periods and decoding of individual stimuli, should be used. This would allow identifying regions that respond selectively to different sound features during their storage in WM.

In summary, the present multivariate fMRI decoding study yielded evidence for a selective processing of pitch and location during an auditory WM task. We identified feature-specific signal patterns in several brain areas including the auditory cortex, regions that are thought to constitute the auditory spatial and non-spatial processing streams, but also in regions not typically associated with auditory processing like the occipital cortex. Further research is needed to determine whether these regions also carry stimulus-selective information during processing in WM and, if this is the case, which regions represent either spatial or non-spatial or both types of content.

## Data Availability Statement

The raw data supporting the conclusions of this article will be made available by the authors, without undue reservation.

## Ethics Statement

The studies involving human participants were reviewed and approved by Ethik-Kommission des Fachbereichs Medizin, Goethe-Universität Frankfurt am Main. The participants provided their written informed consent to participate in this study.

## Author Contributions

SC, CB, and JK conceived and designed the study. ME, SC, CF, and CB collected the data. ME and SC analyzed the data. ME, SC, CF, CB, and JK interpreted the data. ME, SC, and JK prepared the manuscript. All authors contributed to manuscript revision and have read and approved the submitted version.

## Conflict of Interest

The authors declare that the research was conducted in the absence of any commercial or financial relationships that could be construed as a potential conflict of interest.

## Footnotes

1 http://www.nisox.org/Software/SnPM
